# Study of Antibacterial and Anticancer Properties of bioAgNPs Synthesized Using *Streptomyces* sp. PBD-311B and the Application of bioAgNP-CNC/Alg as an Antibacterial Hydrogel Film against *P. aeruginosa* USM-AR2 and MRSA

**DOI:** 10.3390/molecules26216414

**Published:** 2021-10-24

**Authors:** Hemalatha Murugaiah, Chow Lun Teh, Kai Chew Loh, Ahmad Ramli Mohamad Yahya, Nur Asshifa Md Noh, Noor Hana Hanif Abu Bakar, Daruliza Kernain, Rokiah Hashim, Yazmin Bustami

**Affiliations:** 1 School of Biological Sciences, Universiti Sains Malaysia, Gelugor 11700, Malaysia; hemalatha.murugaiah@gmail.com (H.M.); virobbvip2010@gmail.com (C.L.T.); kc960131@gmail.com (K.C.L.); armyahya@usm.my (A.R.M.Y.); nurasshifa@usm.my (N.A.M.N.); 2 School of Applied Sciences, Faculty of Integrated Life Sciences, Quest International University, Ipoh 30250, Malaysia; 3 School of Chemical Sciences, Universiti Sains Malaysia, Gelugor 11700, Malaysia; hana_hanif@usm.my; 4 Institute for Research in Molecular Medicine (INFORMM), Universiti Sains Malaysia, Gelugor 11700, Malaysia; daruliza@usm.my; 5 School of Industrial Technology, Universiti Sains Malaysia, Gelugor 11700, Malaysia; hrokiah@gmail.com

**Keywords:** silver nanoparticles, *Streptomyces* sp., biosynthesis, antibacterial, anticancer, cellulose nanocrystals, alginate, wound healing

## Abstract

Here, we report the extracellular biosynthesis of silver nanoparticles (AgNPs) and determination of their antibacterial and anticancer properties. We also explore the efficacy of bioAgNPs incorporated in cellulose nanocrystals (CNCs) and alginate (Alg) for the formation of an antibacterial hydrogel film. *Streptomyces* sp. PBD-311B was used for the biosynthesis of AgNPs. The synthesized bioAgNPs were characterized using UV-Vis spectroscopy, TEM, XRD, and FTIR analysis. Then, the bioAgNPs’ antibacterial and anticancer properties were determined using TEMA and cytotoxicity analysis. To form the antibacterial hydrogel film, bioAgNPs were mixed with a CNC and Alg solution and further characterized using FTIR analysis and a disc diffusion test. The average size of the synthesized bioAgNPs is around 69 ± 2 nm with a spherical shape. XRD analysis confirmed the formation of silver nanocrystals. FTIR analysis showed the presence of protein capping at the bioAgNP surface and could be attributed to the extracellular protein binding to bioAgNPs. The MIC value of bioAgNPs against *P. aeruginosa* USM-AR2 and MRSA was 6.25 mg/mL and 3.13 mg/mL, respectively. In addition, the bioAgNPs displayed cytotoxicity effects against cancer cells (DBTRG-0.5MG and MCF-7) and showed minimal effects against normal cells (SVG-p12 and MCF-10A), conferring selective toxicity. Interestingly, the bioAgNPs still exhibited inhibition activity when incorporated into CNC/Alg, which implies that the hydrogel film has antibacterial properties. It was also found that bioAgNP-CNC/Alg displayed a minimal or slow release of bioAgNPs owing to the intermolecular interaction and the hydrogel’s properties. Overall, bioAgNP-CNC/Alg is a promising antibacterial hydrogel film that showed inhibition against the pathogenic bacteria *P. aeruginosa* and MRSA and its application can be further evaluated for the inhibition of cancer cells. It showed benefits for surgical resection of a tumor to avoid post-operative wound infection and tumor recurrence at the surgical site.

## 1. Introduction

Silver nanoparticles (AgNPs) have long been known to be a natural antimicrobial agent and have been used since ancient times. They are an effective bactericidal agent that acts on multiple cellular levels and affects several parts of bacteria simultaneously; thus, the tendency of bacteria to develop resistance to AgNPs is presumably low [1,2]. Furthermore, AgNPs also have an excellent anticancer property that exhibits a growth-inhibitory effect on various cancer cells as reported in [3,4,5].

The synthesis of AgNPs using the biological technique biosynthesis is considered to be a better approach to countering limitations to physical and chemical techniques since it is a safe, eco-friendly, and cost-effective technique [6,7,8]. In addition, biosynthesis could produce more stable AgNPs due to the presence of a protein as a natural capping agent [9]. In general, this method takes the green chemistry approach by utilizing the secondary metabolites produced by higher plants, fungi, or bacteria, which act as cell factories for synthesizing nanoparticles. In nature, only a small number of bacteria can survive in a high concentration of silver and synthesize AgNPs [10]. Biosilver nanoparticles (bioAgNPs) have been produced in fungi (*Fusarium oxysporum* and *Aspergillus fumigatus* [11,12]), fruit extracts (*Carica papaya*, *Garcinia mangostana,* and *Vitis vinifera* [13,14,15]), and prokaryotic microorganisms (thermoalkotolerant *Enterobacteria* strains, *Bacillus licheniformis, Streptomyces* sp., and *Aspergillus fumigatus* [16,17,18]).

A recent study reported the potential application of AgNPs in hydrogel wound dressings. Hydrogel wound dressings are seen as an effective alternative wound healing treatment. This type of dressing offers several advantages, including biocompatibility, the provision of hydration and a cool sensation, the efficient absorption of excess exudate, and an accelerated wound-healing process [19]. It has become evident that bacteria in an infected wound become more resistant towards conventional antibiotic treatments since the bacteria produce biofilms, and mature biofilms establish a protective microenvironment [20]. For this reason, AgNPs are perceived as a suitable alternative to inhibit the formation of bacteria due to their antibacterial property and thereby accelerate wound healing. A study conducted by Lustosa’s group used a natural cashew-gum-coated silver nanoparticle (NCG-AgNP) hydrogel and reported that the wound contraction index was significantly higher for male Wistar rats treated with the NCG-AgNP hydrogel as compared with the control group [21]. In another study, Nguyen and coworkers [22] reported that mice with a surgical wound treated with a AgNP-Chitosan/PVA hydrogel showed a higher rate of wound size reduction.

Natural biopolymers, such as alginate, are commonly used for the formation of hydrogels. However, hydrogels formed from alginate alone show poor mechanical strength [23]. The combination of alginate with another reinforcement material, namely nanocellulose, showed a significant improvement in stability, the swelling property, the encapsulation efficiency, and the controlled release of partially attached particles [24]. Nanocellulose is a nanoscopic polysaccharide biopolymer in which the nanocellulose particles are smaller than 1 μm in size [25]. Furthermore, nanocellulose can form mechanically stronger and highly porous bulk materials such as aerogels and films [26]. These highly porous and strong bulk materials can serve as a matrix for the attachment of different nanomaterials, such as metal nanoparticles (e.g., silver nanoparticles (AgNPs) and gold nanoparticles (AuNPs)) [27].

In this study, we performed an extracellular biosynthesis of AgNPs using *Streptomyces* sp. PBD-311B and characterized the synthesized bioAgNPs using UV-Vis, Transmission Electron Microscopy (TEM), Fourier Transform Infrared Spectroscopy (FTIR), and X-ray diffraction (XRD) analyses. Then, the bioAgNPs’ antibacterial and anticancer properties were determined using a tetrazolium microplate assay (TEMA) and cytotoxicity analysis. Finally, we evaluated the inhibition activity of bioAgNPs when incorporated into CNC/Alg for the formation of an antibacterial hydrogel film. The obtained bioAgNP-CNC/Alg hydrogel film was tested against the common wound pathogens *Pseudomonas aeruginosa* and MRSA. This study provides preliminary findings for the application in wound healing of the bioAgNP/CNC/Alg hydrogel film, which will probably be useful for application in the surgical resection of a tumor where the proliferation of cancer cells needs to be inhibited at the surgical site.

## 2. Results and Discussion

### 2.1. Extracellular Biosynthesis of bioAgNPs

**Figure 1:**
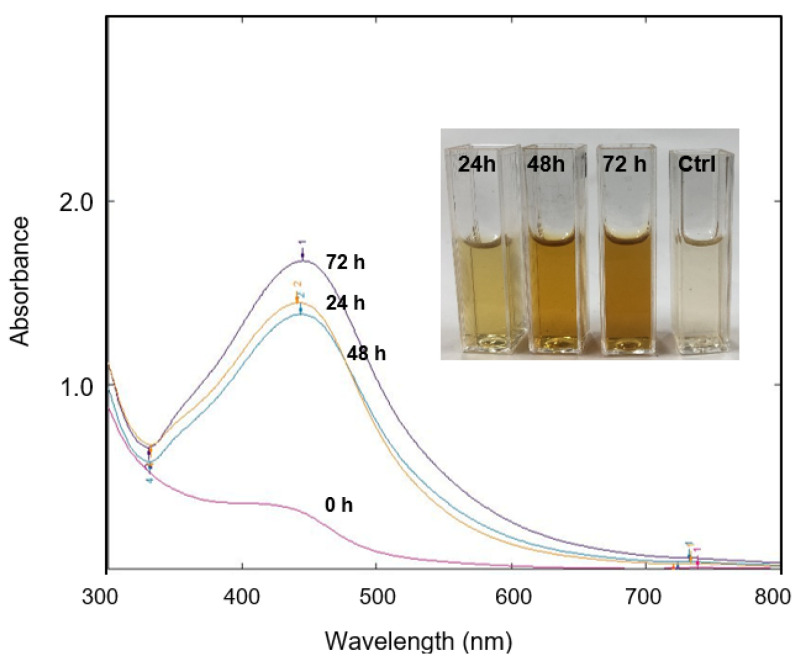
Observation of the LSPR band at different incubation times (0 h (nil), 24 h (441 nm), 48 h (443 nm), and 72 h (445 nm)) confirmed the formation of bioAgNPs. Inset: color changes after incubation for 24 h (light yellow), 48 h (light brown), and 72 h (brown). No color change was observed for the control solution after 72 h of incubation.

The presence of a color change in the solution mixture (AgNO_3_ and *Streptomyces* sp. PBD-311B supernatant) indicated the formation of bioAgNPs. After 72 h of incubation, the solution’s color intensified from light yellow to brownish (Figure 1, inset) as compared with the control solution (without the addition of AgNO_3_). In general, AgNPs were easy to identify by the formation of the brown color. Furthermore, the observation of a strong localized surface plasmon resonance (LSPR) band at ~440 nm, as characterized by UV-Vis spectroscopy, confirmed the successful formation of bioAgNPs (Figure 1). Typically, AgNPs exhibit a LSPR in the range of 400–500 nm [28]. As reported in several studies, the absorption spectra directly reflect the size and morphology of AgNPs [2,29,30,31,32].

The synthesized bioAgNPs taken at different incubation times (24 h, 48 h, and 72 h) showed a slight red-shift towards longer wavelengths (441 nm to 445 nm), which further indicates a slight size change. However, the changes were negligible and we postulated that the presence of secondary metabolites in the solution medium produced stable bioAgNPs after 24 h of incubation. In this study, we used *Streptomyces* sp. PBD-311B, which was isolated from a mangrove area and is well-adapted to the type of extreme environment that could possibly produce unusual metabolites [33]. Therefore, we envisaged that the secondary metabolites secreted by the salt-tolerant *Streptomyces* sp. PBD-311B would display an exceptional bioreduction of the metal salt (AgNO_3_) to bioAgNPs. However, the biosynthesis of nanoparticles is highly dependent on the species and microorganism strain. A study reported that the biosynthesis process of AgNPs involves a reductase enzyme that exists inherently in *Streptomyces* sp. [18]. Alani’s group [18] suggested that *Streptomyces* sp. might produce a higher concentration of, and a different, reductase enzyme than *Aspergillus fumigatus* and the formation of AgNPs depends solely on the intracellular component. However, the authors of [34] successfully synthesized AgNPs extracellularly by using *Streptomyces hygroscopicus* supernatant. Similarly, Bhainsa’s group discovered the capabilities of *Aspergillus fumigatus* in the extracellular biosynthesis of AgNPs [12]. The use of extracellular biosynthesis to produce bioAgNPs provides a great solution for the purification process. It is beneficial for scaling up the production of bioAgNPs and offers a rapid, simple, and cost-effective technique since a minimal number of purification steps is required.

### 2.2. Characterization of the Shape, Size, and Crystallinity of the bioAgNPs

**Figure 2:**
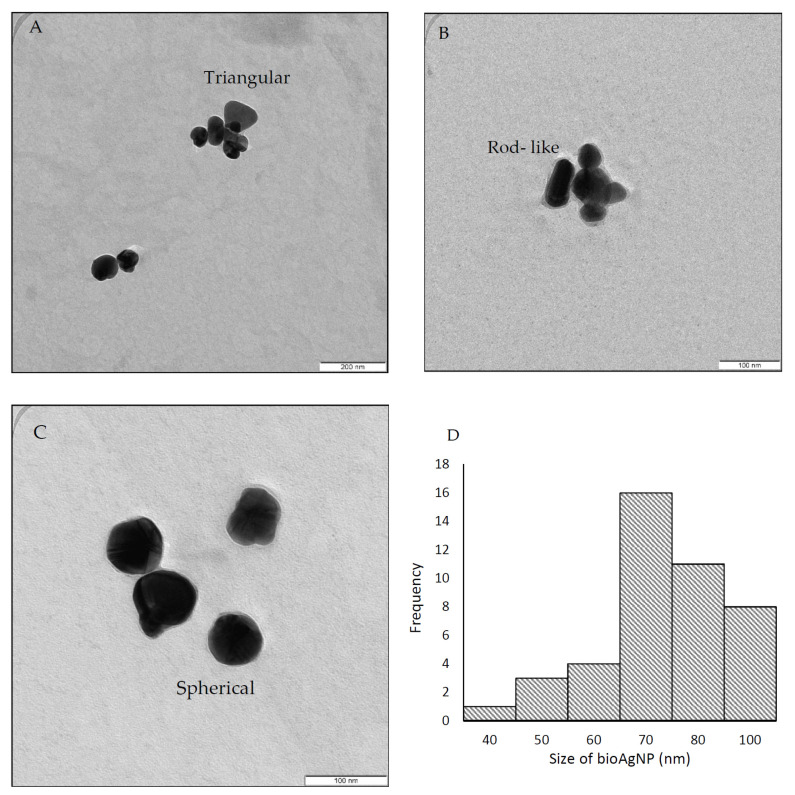
TEM analysis revealed the different shapes of synthesized bioAgNPs: triangular (**A**), rod-like (**B**), and spherical (**C**). The broad size distribution ranging from 40 to 100 nm (**D**).

As shown in Figure 2A–C, the TEM images reveal the different shapes of the bioAgNPs: triangular, rod-like, and spherical. According to [8], the synthesis of AgNPs using a bioreducing agent would form irregularly shaped nanoparticles. Factors such as the biochemical and genetic nature of the microbial strains primarily control the morphology of nanoparticles [35]. However, the majority of the bioAgNPs synthesized in this work appeared to be spherical in shape at around 445 nm. The authors of [30] produced spherical AgNPs within 450 nm. Figure 2D shows a broad size distribution, ranging from 40 to 100 nm, with an average particle size of approximately 69 ± 2 nm. A similar finding was reported by Karthik and coworkers, where AgNPs synthesized using *Streptomyces* sp. LK3 supernatant produced AgNPs in the size range of 0.5–100 nm [33].

**Figure 3:**
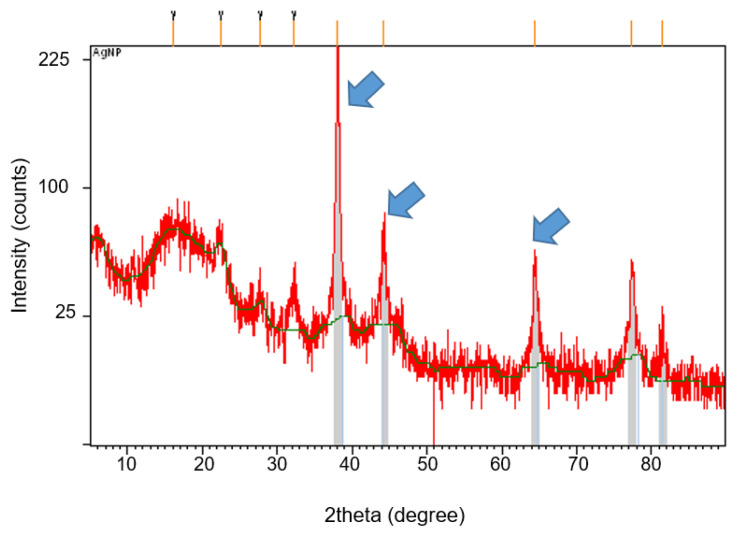
XRD analysis of bioAgNPs synthesized by *Streptomyces* sp. PBD-311B supernatant. Arrows indicate the main diffraction peaks of bioAgNPs positioned at 38.1°, 44.2°, and 64.4°.

As shown in Figure 3, the crystalline nature of the synthesized bioAgNPs was successfully determined using XRD analysis. The main diffraction peaks at the 2θ position, 38.1°, 44.2°, and 64.4°, correspond to (1 1 1), (2 0 0), and (2 2 0), respectively. This Bragg’s reflection is based on the face-centered cubic (fcc) structure of silver [36]. The highest diffraction peak occurred at 38.1°, which confirms that the synthesized bioAgNPs are silver nanocrystals and is in agreement with other reported studies [18,34,37]. In addition, several unassigned peaks were also observed in the vicinity of the characteristic peaks and might indicate the presence of an organic compound (a protein or other biomolecule) that acts as a capping agent to stabilize the bioAgNPs, since proteins are commonly reported to cap the AgNP surface [9,18].

### 2.3. Antibacterial Activity of bioAgNPs

**Figure 4:**
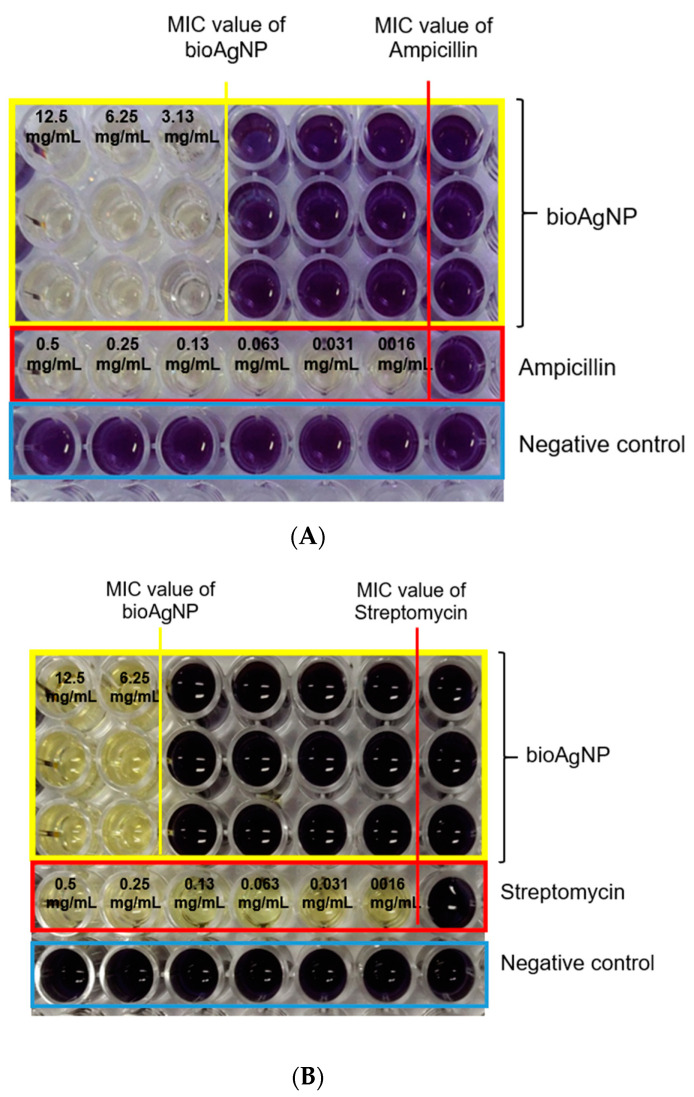
Observation of color changes using TEMA of bioAgNPs and commercial antibiotics against MRSA (**A**) and *P. aeruginosa* USM-AR2 (**B**). The MIC value is denoted as the lowest concentration of the tested samples. Lines 1–3 show the experiment performed in triplicate on the bioAgNPs. Line 4 shows the commercial antibiotics used (ampicillin and streptomycin). Line 5 shows the negative control.

The antibacterial activity of the bioAgNPs against *P. aeruginosa* USM-AR2 and MRSA was confirmed using TEMA. A notable inhibition of growth can be observed in Figure 4. The wells with the purple formazan color indicate the growth of bacteria, whereas the well with the yellow tetrazolium color indicates no bacterial growth. The lowest concentration of the tested agent that showed no visible growth of bacteria is marked as the MIC value. The MIC values for bioAgNPs against MRSA and *P. aeruginosa* USM-AR2 are 3.13 mg/mL and 6.25 mg/mL, respectively (Table 1). On the other hand, streptomycin and ampicillin showed much lower MIC values since these commercial antibiotics are designed to exhibit a specific attack against bacteria, while bioAgNPs attack bacteria in a non-specific fashion; thus, a large difference in the MIC values is visible [23,38,39,40]. However, there is a growing concern about the resistance of *P. aeruginosa* and MRSA to a wide range of antibiotics. In 2017, the World Health Organization (WHO) published a list of antibiotic-resistant bacteria and *P. aeruginosa* was listed in the critical-priority group, while MRSA was listed in the high-priority group [36]. Thus, bioAgNPs show potential as an antibacterial agent to combat the multi-drug resistance (MDR) problem.

The bioAgNPs produced in this work showed a fairly broad size distribution (40–100 nm), consequently conferring a high MIC value. In addition, the average size of the bioAgNPs is 69 ± 2 nm. These nanoparticles might exhibit low inhibition efficiency since it has been reported that AgNPs smaller than 50 nm in size exhibit an effective antimicrobial property [40]. The authors of [41] reported that AgNPs produced by *Streptomyces xinghaiensis* OF_1_ with a size ranging between 5 and 20 nm exhibited a satisfying MIC value of 16 μg/mL and 256 μg/mL against *P. aeruginosa* and *S. aureus*, respectively. Furthermore, the release of silver ions is size-dependent and, according to [42], AgNPs smaller than 20 nm in size release 100 times more silver ions than bulk silver particles. As shown in Figure 4 and Table 1, bioAgNPs showed better inhibition against the Gram-positive bacterium MRSA than against the Gram-negative bacterium *P. aeruginosa* USM-AR2. This phenomenon is probably due to the difference in the membrane cell structure. The inhibition mechanism was further observed using TEM image analysis.

**Figure 5:**
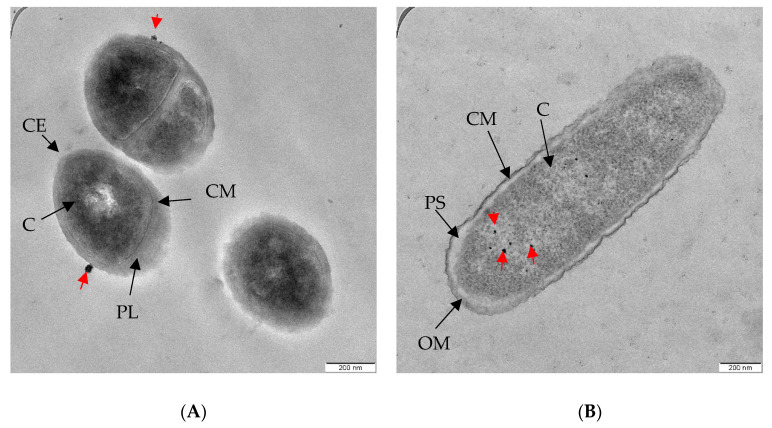
TEM images show bioAgNPs (**A**) that accumulated on the clinical MRSA envelope, and (**B**) were internalized in the *P. aeruginosa* USM-AR2 cytoplasm. Red arrows indicate the presence of bioAgNPs. CE, cell envelope; PL, peptidoglycan layer; CM, cytoplasmic membrane; C, cytoplasm; OM, outer membrane; PS, periplasma space.

Based on Figure 5, bioAgNPs interact with and invade both types of bacteria differently. As can be observed in Figure 5A, most of the bioAgNPs were found to have accumulated on the MRSA envelope. Work by Foxley and co-workers provided an insight into the strong interaction between the wall teichoic acid (WTA), an anionic phosphate group that is found in abundance on the MRSA envelope, and the branched poly(ethylenimine) (BPEI), a polycation [43]. They also reported that BPEI is located in the cell wall and not in the cytoplasm. Accordingly, we suggest that a similar electrostatic interaction occurred between bioAgNPs and WTA on the MRSA envelope. This interaction might affect the overall charge of the cell wall and interrupt the normal biosynthesis of WTA. It further weakened the peptidoglycan linkages, which subsequently leaked intracellular content, a phenomenon known as ‘pitting’ [44].

BioAgNPs were mostly found in the cytoplasm of *P. aeruginosa* USM-AR2, which is easy to penetrate (Figure 5B). Similar conditions were observed in our previous study [45]. The Gram-negative *P. aeruginosa* cell wall is composed of lipopolysaccharides (LPS) with a highly negative charge that promotes the adhesion of AgNPs [46]. In addition, internalization of bioAgNPs into Gram-negative bacteria is seen as effective due to the different membrane structure. It is assumed that the internalization of bioAgNPs initiates a cascade of bacterial reactions, including the binding of bioAgNPs to various bacterial organelles, leading to a disruption of the respiratory chain and disorders in cell division that eventually cause cell death [47].

### 2.4. Cytotoxicity Analysis of bioAgNPs

**Figure 6:**
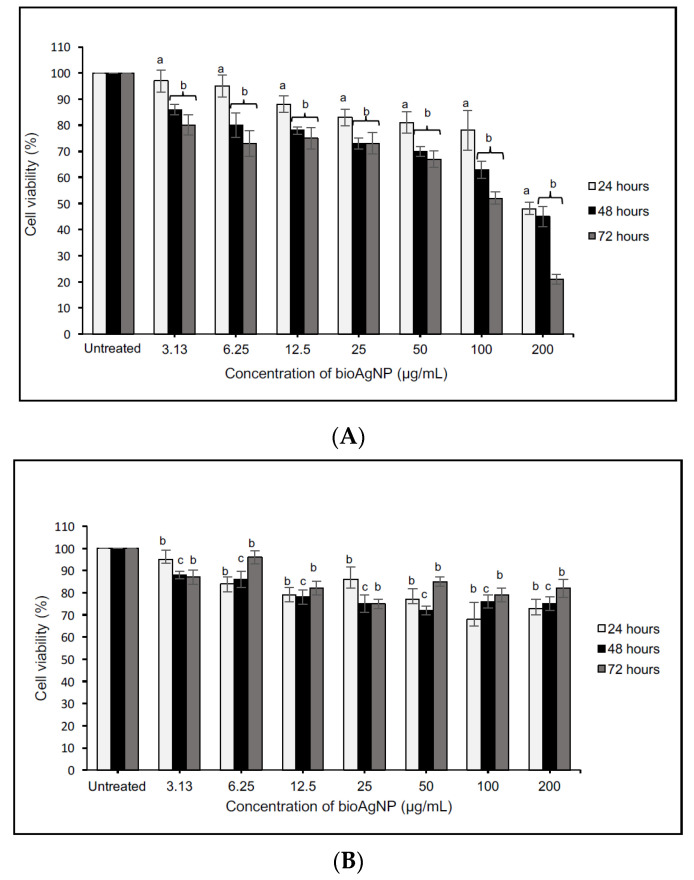
Cytotoxicity analysis of various concentrations of bioAgNPs against DBTRG-0.5MG (**A**) and SVG-p12 (**B**) cells within 24–72 h of treatment. The values presented are the means ± S.E. from three independent experiments. Statistical analysis was performed using Student’s *t*-test with ^a^ *p* < 0.05, ^b^ *p* < 0.01, and ^c^ *p* < 0.001 significantly different to the untreated cells.

**Figure 7:**
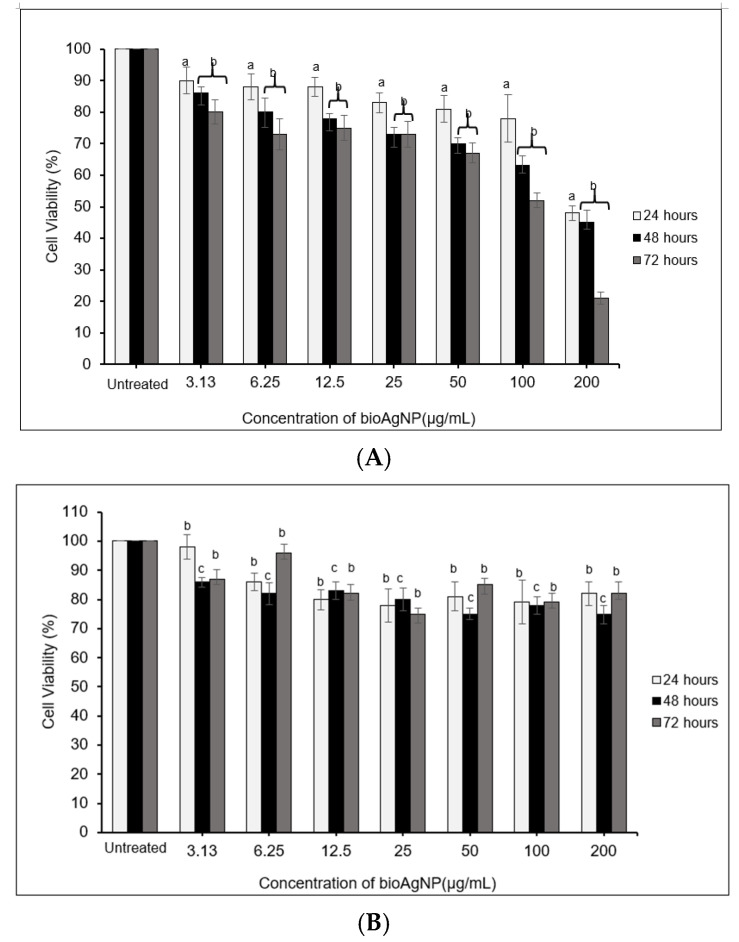
Cytotoxicity analysis of various concentrations of bioAgNPs against MCF-7 (**A**) and MCF-10A (**B**) cells within 24–72 h of treatment. The values presented are the means ± S.E. from three independent experiments. Statistical analysis was performed using Student’s *t*-test with ^a^ *p* < 0.05, ^b^ *p* < 0.01, and ^c^ *p* < 0.001 significantly different to the untreated cells.

Figure 6 and Figure 7 present the evaluation of varying concentrations of bioAgNPs (3.13–200 μg/mL) against a human glioblastoma cell line (DBTRG-0.5MG), a normal brain cell line (SVG-p12), a breast cancer cell line (MCF-7), and a normal breast cell line (MCF-10A). Based on the evaluation, a similar trend was observed for both cancer cells and was significantly different as compared with the untreated cells. These results suggest that an increase in the bioAgNP concentration reduced the cell viability or inhibited the cell proliferation, confirming the pro-apoptotic potential of the bioAgNPs. The cytotoxicity responded in a dose-dependent manner and similar findings can be found in other studies [4,48,49]. Importantly, the cytotoxicity effects were profoundly significant when the bioAgNPs were incubated for 48 to 72 h (^b^ *p* < 0.01). The LC_50_ values recorded for DBTRG-0.5MG and MCF-7 are similar (see Table 2) The lowest LC_50_ value was observed after 72 h of treatment, which implies greater cytotoxic effects.

Interestingly, the dose-dependent analysis performed against normal cells (SVG-p12 and MCF-10A) showed very minimal cytotoxic effects since ~80% of the cells remained viable even after being treated with the highest concentration (200 μg/mL). For this reason, the LC_50_ value for the normal cells could not be determined. This indicates that the bioAgNPs displayed an anticancer property with selective toxicity. Eid and co-workers showed that biogenic AgNPs demonstrate selective toxicity, where the anti-proliferative effect is more significant on cancer cells than on normal cells [3].

### 2.5. Formation of CNC/Alg and bioAgNP/CNC/Alg Films

**Figure 8:**
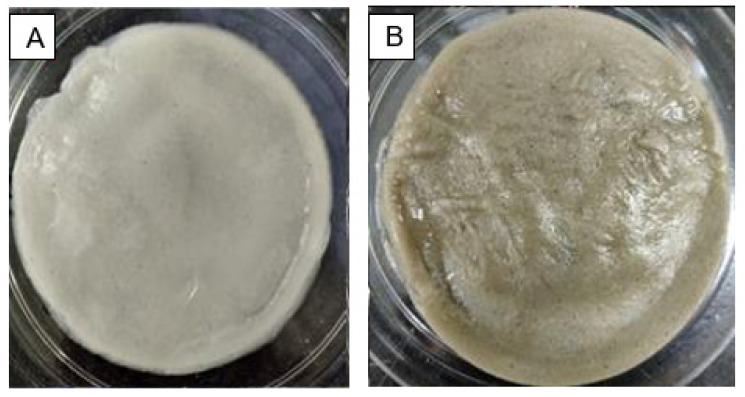
The formation of two types of hydrogel film in the petri dish and a comparison of the physical morphology of the CNC/Alg film (**A**) and the bioAgNP/CNC/Alg film (**B**).

As depicted in Figure 8, CNC/Alg and bioAgNP/CNC/Alg hydrogel films were successfully formed with a thickness of approximately 3 mm and a rigid structure. The brown coloration of bioAgNP/CNC/Alg (Figure 8B) provided an early indication that bioAgNPs had been successfully incorporated into the CNC/Alg hydrogel. Further analysis was conducted using FTIR to observe the interaction between bioAgNPs and the CNC/Alg hydrogel film in detail.

**Figure 9:**
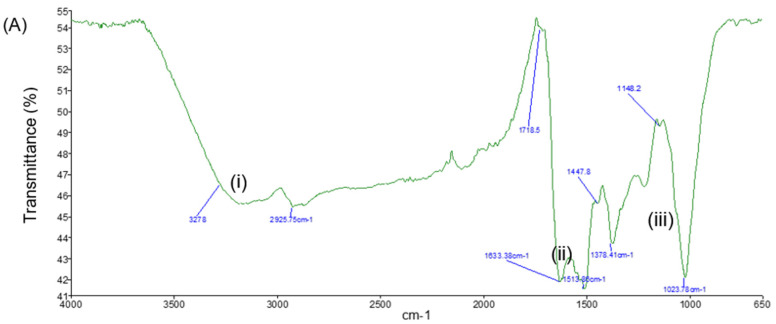

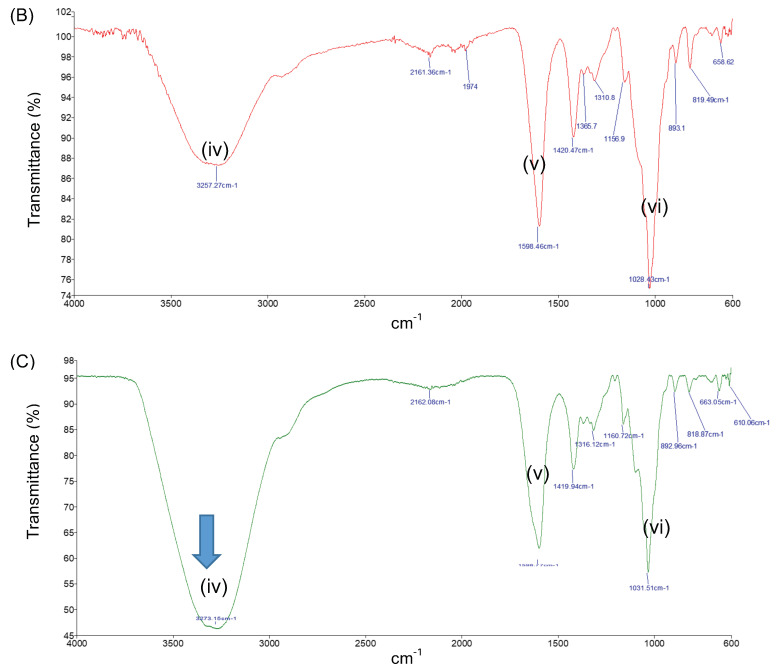
FTIR analysis revealed several characteristic peaks of bioAgNPs (**A**), CNC/Alg (**B**), and bioAgNP/CNC/Alg (**C**). The main characteristic peaks are labeled (i), (ii), (iii), (iv), (v), and (vi).

FTIR analysis was conducted for the bioAgNPs, the CNC/Alg hydrogel film, and the bioAgNP/CNC/Alg hydrogel film. In Figure 9A, several signature peak regions can be observed for the synthesized bioAgNPs. The broad peak at 3278 cm^−1^ (i) indicates the N-H functional groups of the protein and the O-H stretching vibration of the alcohol or phenol [50]. The peak at 1633 cm^−1^ (ii) represents the amide I with a beta sheet structure that occupies the area near 1632 cm^−1^. Furthermore, the peaks at around 1543 cm^−1^ and 1022 cm^−1^ (iii) indicate the C-C stretching of the aromatic compound and the CHO stretching vibration of amine groups, respectively [51]. Therefore, it is evident that the nanoparticles were mostly stabilized by the protein secreted by the biomass [50,52,53]. This finding supports the presence of protein capping on the bioAgNP surface, suggesting that it performs the function of stabilizing bioAgNPs in an aqueous environment. As reported in [35], the isolated marine bacterium *Streptomyces albidoflavus* CNP10 produces an extracellular protein compound that can bind to synthesized AgNPs through free amine groups as well as cysteine residues where the amine (N-H) stretching vibration band and the amide (N-H) bending vibration are present.

Figure 9B,C present the characteristic peaks for the CNC/Alg and bioAgNP/CNC/Alg hydrogel films. The O–H stretching vibration band shown at around 3250 cm^−1^ to 3700 cm^−1^ (iv) suggests that the hydroxyl group (–OH) is the main functional group present in the nanocellulose and alginate biopolymer [54,55]. In addition, this suggests that the interaction between CNC and Alg occurred through hydrogen bonding [56]. After bioAgNPs were incorporated into the hydrogel film, the vibration intensity of most of the characteristic peaks increased. It is also important to note that the amide bending vibration (ii and iii) for bioAgNPs disappeared, which might indicate that the bioAgNPs favored the strong intermolecular interaction with the CNC/Alg film. As mentioned previously, the hydroxyl group is the main functional group present in the CNC/Alg film. The intense band found at 3273 cm^−1^, as indicated by the arrow, suggests that the interaction might have occurred through hydroxyl groups with Ag through oxygen.

In addition, the characteristic peaks at 1598.5 cm^−1^ and 1420.5 cm^−1^ were slightly shifted to 1598.8 cm^−1^ and 1419.9 cm^−1^, respectively (v). The peaks generated approximately at 1594 cm^−1^ and 1410 cm^−1^ are characteristic of the asymmetric and symmetric elongation of carboxylate groups of the alginate polymer [51,57], thus suggesting the successful formation of ionic crosslinking between the Ca^+2^ of the carboxylate group and the (-COO^−^) of the alginate. Other peaks generated between 600 cm^−1^ and 1200 cm^−1^ (vi) indicate several stretching vibrations, such as C–H, O–H, C–O, and C–O–C, of the glycosidic ring of the polysaccharide structure [57].

### 2.6. Application of bioAgNP/CNC/Alg Hydrogel Film

**Figure 10:**
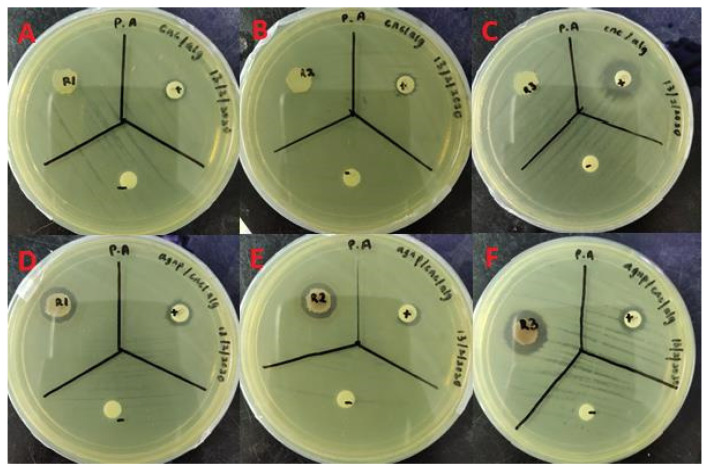
Disc diffusion test of the CNC/Alg hydrogel film (**A**–**C**) and the bioAgNP-CNC/Alg hydrogel film (**D**–**F**) against *P. aeruginosa* USM-AR2. BioAgNP-CNC/Alg and CNC/Alg are denoted (R), streptomycin is denoted (+), and dH_2_O is denoted (−). This test was conducted in triplicate.

**Figure 11:**
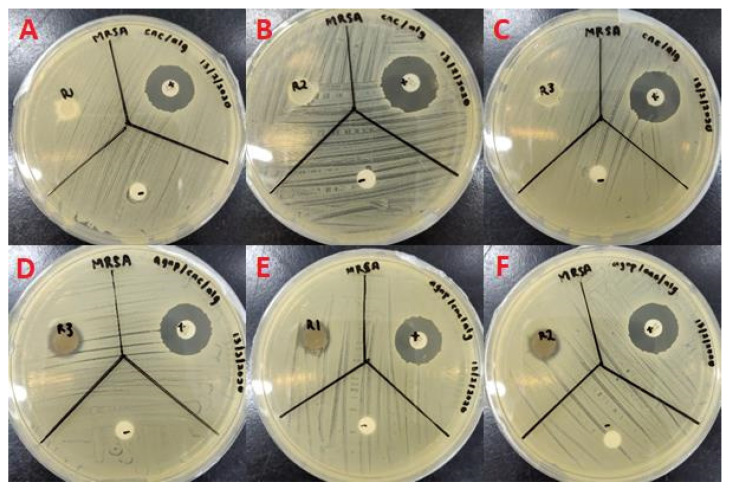
Disc diffusion test of the CNC/Alg hydrogel film (**A**–**C**) and the bioAgNP-CNC/Alg hydrogel film (**D**–**F**) against MRSA. BioAgNP-CNC/Alg and CNC/Alg are denoted (R), vancomycin is denoted (+), and dH_2_O is denoted (−). This test was conducted in triplicate.

In Figure 10 and Figure 11, inhibition zones can clearly be observed for the bioAgNP/CNC/Alg hydrogel film against both *P. aeruginosa* USM-AR2 and MRSA, which indicate successful antibacterial effects. In contrast, no inhibition zone can be observed for the CNC/Alg hydrogel film, which signifies that bioAgNPs mainly contribute to the antibacterial property of the hydrogel film. The difference in inhibition is statistically significant between the tested compounds (bioAgNP/CNC/Alg, CNC/Alg, the antibiotics (+), and the negative control (−)) (*p* < 0.05). In Table 3, the measured diameter for the bioAgNP/CNC/Alg hydrogel film against *P. aeruginosa* USM-AR2 (13 ± 0.7 mm) is slightly larger compared with MRSA (11 ± 0 mm), but the difference is not significant (Student’s *t*-test, *p* > 0.05). Notably, the bioAgNP/CNC/Alg hydrogel film displayed the minimum inhibition efficiency for both types of bacteria, which might be due to the minimal or slow release of bioAgNPs. Owing to the strong intermolecular interaction of bioAgNPs in the crosslinked hydrogel film, as explained in the discussion of the FTIR analysis, a minimal amount of bioAgNPs might have been released. Additionally, hydrogels ensure the slow release of AgNPs as they have hollow pores, a large number of water molecules, and a variety of functional groups [58]. Thus, further study to determine the sufficient release of bioAgNPs from the hydrogel film to confer better antibacterial activity is recommended. Surprisingly, streptomycin (+) showed much a smaller inhibition zone (10 ± 0.9 mm) with an inconsistent diameter size against *P. aeruginosa* USM-AR2. It is evident that most of the antibiotic-resistant species isolated from clinical samples have demonstrated a significance increase in antibiotic resistance [59]. However, when tested using TEMA, streptomycin showed a better MIC value, confirming the efficacy of streptomycin against *P. aeruginosa* USM-AR2.

In general, bioAgNP/CNC/Alg is a promising antibacterial hydrogel film and might have potential in wound healing treatments, especially in the treatment of wounds chronically infected with pathogenic bacteria such as *P. aeruginosa* and *S. aureus.* These bacteria are known to be common wound pathogens [60]. Recently, there has been growing interest in developing hydrogels with antibacterial and anticancer properties that can expedite the wound healing process. These multifunctional properties are important to the surgical resection of a tumor [61]. In the present study, bioAgNPs demonstrated anticancer activity against different cell lines. Interestingly, they also exhibited selective toxicity, which might prevent toxic effects associated with Ag. Therefore, we propose that bioAgNPs incorporated in a CNC/Alg hydrogel film can be used for the surgical resection of a tumor. This might be beneficial for the prevention of post-operative wound infections and may help to prevent tumor recurrence by inhibiting the proliferation of cancer cells at the surgical site [61].

## 3. Materials and Methods

### 3.1. Materials

#### 3.1.1. Chemical Reagents

Silver nitrate (AgNO_3_) and potassium hydroxide (MW, 56.11 g/mol) were purchased from R&M Chemicals (Selangor, Malaysia). Yeast extract, glucose, and malt extract were purchased from Friendemann Schmidt (Western Australia, Australia). Mueller–Hinton (MH) powder, vancomycin, and streptomycin antimicrobial susceptibility discs were purchased from Oxoid (UK). Sodium alginate (C_6_H_9_NaO_7_) (MW, 216.12 g/mol), 3-(4,5-dimethyl-2-thiazolyl)-2,5-diphenyltetrazolium bromide (MTT) powder, Tween-80, dimethyl sulfoxide (DMSO), phosphate-buffered saline (PBS) tablets, fetal horse serum (FHS), insulin, hydrocortisone, epidermal growth factor (EGF), and penicillin–streptomycin (penstrep) were purchased from Sigma-Aldrich (St. Louis, MO, USA). Roswell Park Memorial Institute (RPMI) 1640 media, high-glucose Dulbecco’s Modified Eagle’s Medium (DMEM), and fetal bovine serum (FBS) were purchased from Gibco. Calcium chloride (CaCl2) (MW, 110.98 g/mol) was purchased from Bendosen Laboratory Chemicals (Kuala Lumpur, Malaysia). Microbial solution media, including 0.5 McFarland standard, 0.85% NaCl solution, Luria–Bertani (LB) powder, streptomycin, and ampicillin were obtained from the School of Biological Sciences, USM, Penang, Malaysia. Oil palm trunk powder was obtained from the School of Industrial Technology, USM, Penang, Malaysia.

#### 3.1.2. Bacterial Strains and Human Cell Lines

For the synthesis of bioAgNPs, an actinobacterium was used. This actinobacterium was isolated from a mangrove area at Balik Pulau, Penang, Malaysia and was identified as *Streptomyces* sp. PBD-311B [62]. It was cultivated in ISP-2 media at 30 °C. ISP-2 media contain malt extract (10 g/L), glucose (4 g/L), and yeast (4 g/L) and the solution was adjusted to a pH of 7. For antibacterial testing, *Pseudomonas aeruginosa* USM-AR2 isolated from a crude oil sample was used [63]. The clinical methicillin-resistant *Staphylococcus aureus* (MRSA) was obtained from the Advanced Medical and Dental Institute (IPPT), Universiti Sains Malaysia. All bacterial strains were obtained from the School of Biological Sciences, Universiti Sains Malaysia, Penang. For the cytotoxicity analysis, human glioblastoma cells (DBTRG-0.5MG), normal brain cells (SVG p12), breast cancer cells (MCF-7), and normal breast cells (MCF 10A) were obtained from Dr. Daruliza’s lab at the Institute for Research in Molecular Medicine (INFORMM), Universiti Sains Malaysia, Penang.

### 3.2. Methods

#### 3.2.1. Cultivation of *Streptomyces* sp. PBD-311B

The fermentation media and conditions were adapted as in [64] with several modifications. First, glycerol stock of *Streptomyces* sp. PBD-311B was propagated in 50 mL of ISP-2 media at 200 rpm for 4 days. Then, the bacterial culture was homogenized using a blender before being centrifuged at 14,000× *g* for 10 min followed by resuspension in distilled water. About 10% (*v*/*v*) of the inoculum was transferred into fresh ISP-2 media (pH 7) with a total working volume of 50 mL and incubated in a rotary shaker for 4 days at 200 rpm and RT. After that, the culture was centrifuged at 2700× *g* for 20 min at 11 °C. Finally, only the cell-free supernatant was collected and filter-sterilized using a 0.22 μm polyethersulfone (PES) filter, while the cell pellet was discarded.

#### 3.2.2. Extracellular Biosynthesis of AgNPs

The method for the extracellular biosynthesis of AgNPs was adapted from [6] with several modifications. About 50 mL of filter-sterilized cell-free supernatant was mixed with 50 mL of 6 mM AgNO_3_ (1:1 (*v*/*v*)) in a 250 mL Erlenmeyer flask. The control solution was prepared by mixing 50 mL of cell-free supernatant with 50 mL of dH_2_O using a similar ratio. The flasks were wrapped with aluminum foil and agitated at 200 rpm and RT. At different incubation times (0 h, 24 h, 48 h, and 72 h), the synthesized bioAgNPs and the control solutions were subjected to UV-Vis spectroscopy analysis. Only the bioAgNP solution incubated for 72 h was used in further analyses. The sample was transferred to a sterile Falcon tube and stored in the dark for at least 12 h at −40 °C, then was subjected to a freeze-drying process for 3 days. About 25 mg of the obtained crude powder was dissolved in 1 mL of sterile dH_2_O and sonicated for 1 h before being filter-sterilized with a 0.22 μm PES filter. The sterile bioAgNP stock solution (25 mg/mL) was wrapped in aluminum foil at RT until use.

#### 3.2.3. UV-Vis Spectroscopy Analysis of bioAgNPs

UV-Vis spectroscopy was conducted to observe the localized surface plasmon resonance (LSPR) of the bioAgNPs. About 0.5 mL of bioAgNP solution was added to 2.5 mL of DI H_2_O. The color changes in the bioAgNP aliquots and the control aliquots (containing only cell-free supernatant) were measured. The wavelength was set in the range of 300 to 800 nm at a resolution of 1 nm and the analysis was conducted using a Shimadzu spectrophotometer (Shimadzu UV-1800, Kyoto, Japan).

#### 3.2.4. Transmission Electron Microscopy (TEM) Analysis

TEM analysis was conducted at the Electron Microscope Unit, School of Biological Sciences, Universiti Sains Malaysia. A drop of bioAgNP stock solution was used as-is and was placed on top of the rough side of a 3 mm carbon-coated copper grid and left to air-dry. The excess moisture was removed by blotting the grid on filter paper before the grid was loaded into the TEM (Zeiss Libra 120, Oberkochen, Germany). The size of bioAgNPs was measured using cellSens Standard Imaging Software (Olympus, Tokyo, Japan).

#### 3.2.5. X-ray Diffraction (XRD) Analysis

The sample was sent to the X-ray Crystallography Laboratory at the School of Physics, Universiti Sains Malaysia. For the XRD analysis, bioAgNP stock solution was dried at 30 °C to obtain a crude powder. This analysis was recorded using a Bruker X-ray diffractometer in the 2θ range of 25° to 90° with Cu Kα radiation and a wavelength of 1.54 Å. The applied voltage was 40 kV and the current used was 30 mA.

#### 3.2.6. Antibacterial Testing Using the Tetrazolium Microplate Assay (TEMA)

The TEMA utilized a colorimetric assay to determine the minimum inhibitory concentration (MIC) value of bioAgNPs against *P. aeruginosa* USM-AR2 and MRSA. For this method, we followed our previous study [45] with several modifications. A loopful of bacteria was grown overnight in Mueller–Hinton (MH) broth. Then, the inoculum was transferred into 5 mL of 0.85% sterile NaCl. The turbidity of the bacterial suspension was adjusted using 0.5 McFarland solution (~10^8^ colony-forming unit (CFU)/mL). After that, the bacterial suspension was further diluted to ~10^5^ CFU/mL. A two-fold serial dilution was carried out using 100 μL of bioAgNP solution (25 mg/mL) and 100 μL of sterile dH_2_O. For antibiotics, 100 μL of streptomycin (1 mg/mL) was added against *P. aeruginosa* USM-AR2, while 100 μL of ampicilin (1 mg/mL) was added against MRSA. Previously, 1 mg of streptomycin and 1 mg of ampicilin were prepared in 1 mL of 1% DMSO solution and filter-sterilized using a 0.22 μm PES filter. Then, a microtiter plate was inoculated with 100 μL of bacterial suspension per milliliter of nutrient broth, homogenized, and incubated at 37 °C. Finally, color changes were observed upon incubation with 50 μL of MTT reagent. MTT is a yellow tetrazolium salt that is converted to a purple formazan by dehydrogenases produced by live cells.

#### 3.2.7. Observation of bioAgNPs’ Inhibition Mechanism Using TEM

The method used was adapted from [65] with a few modifications. A loopful of bacterial suspension incubated overnight, *P. aeruginosa* USM-AR2, and MRSA (concentration, 10^8^ CFU/mL) were inoculated in a mixture of 0.5 mL of bioAgNP solution and 0.5 mL of LB broth (1:1) (*v*/*v*). The mixture was incubated at 37 °C with shaking at 200 rpm for about 6 h before being subjected to centrifugation at 14,000× *g* for 20 min. The obtained bacterial pellet was washed once with 0.1 M PBS before being centrifuged again. The supernatant was discarded, and the cell pellet was soaked in McDowell Trump fixative for at least 2 days. This fixative solution was prepared by mixing formaldehyde and glutaraldehyde (4:1). After that, the soaked cell pellet was centrifuged and washed with 0.1 M PBS twice. Then, the pellet was re-suspended in 1% osmium tetraoxide and left for 1 h under the fume-hood. The stained cells were impregnated in resin and further incubated for 1 week. Then, the resin was sliced using a microtome machine and the cross-sectioned bacterial cells treated with bioAgNPs were observed using TEM.

#### 3.2.8. Cytotoxicity Analysis of bioAgNPs

This analysis followed the method described in [4] with several modifications and was conducted at the Institute for Research in Molecular Medicine (INFORMM), USM, Penang. DBTRG-0.5MG and SVG-p12 were cultured in RPMI 1640, while MCF-7 and MCF-10A were cultured in high-glucose DMEM. All media were supplemented with 10% FBS and 1% penstrep. For MCF-10A, the medium was supplemented with 10% FHS and the following additives: 10 μg/mL insulin, 0.5 μg/mL hydrocortisone, 20 μg/mL EGF, and 1% penstrep. About 100 μL of suspension containing 1 × 10^4^ cells were seeded in a 96-well microtiter plate (BD, USA) and maintained overnight at 37 °C with 5% CO_2_ to reach 80–90% confluence. The next day, the media were carefully discarded and rinsed properly with PBS solution. About 90 μL of fresh media was added to each respective well and treated with various concentrations of bioAgNPs to a final volume of 100 μL per well. Subsequently, the microtiter plate was incubated for 24, 48, and 72 h at 37 °C with 5% CO_2_. After the incubation period, the medium in the wells was discarded and rinsed properly with PBS solution. About 100 μL of fresh media was re-added to each well and 10 μL of WST-1 cell proliferation reagent was added to each well. Then, the microtiter plate was incubated at 37 °C for 2 h with 5% CO_2_. Finally, the cell viability was measured using an ELISA reader (Multiskan Spectrum) at 450 nm. The absorbance reading was carried out in triplicate.

#### 3.2.9. Preparation of Cellulose Nanocrystals (CNCs) from Oil Palm Trunk

Cellulose nanocrystals (CNCs) were extracted from a vascular bundle of oil palm trunk (*Elaeis guineensis*) using the acid hydrolysis technique described in [66]. The extraction process was conducted at the School of Industrial Technology, USM, Penang. The extracted CNCs were homogenized, sonicated, and kept at −20 °C in a fridge overnight before being subjected to a freeze-drying process for 5 days. The freeze-dried CNCs were ground into a fine powder and stored at RT until use.

#### 3.2.10. Formation of the bioAgNP/CNC/Alg Hydrogel Film

**Figure 12:**
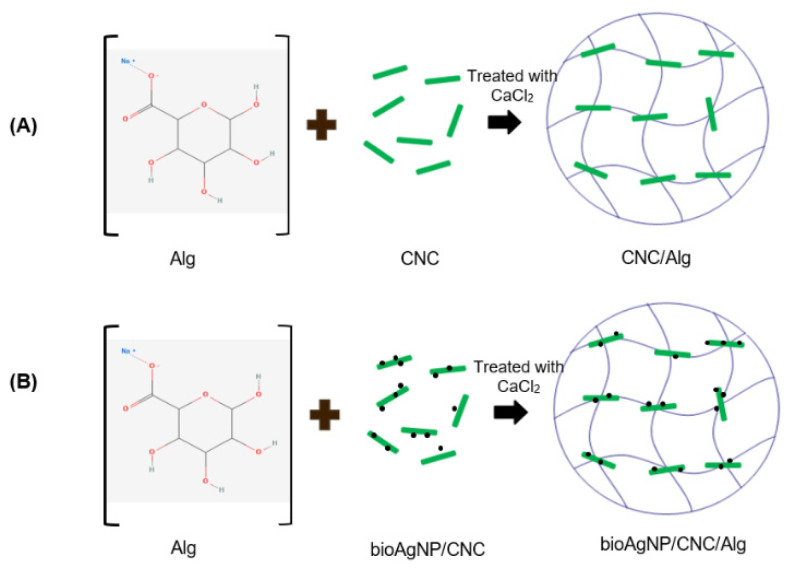
Schematic diagram of the formation of CNC/Alg (**A**) and bioAgNP/CNC/Alg (**B**). This hydrogel film was formed using a heating and stirring technique and was further crosslinked using Ca^2+^ ions.

In a separate beaker, 0.3 g of CNC powder was added to 15 mL of dH_2_O and homogenized for 5 min, while 0.15 g of alginate (Alg) was added to 10 mL of dH_2_O and pre-heated and stirred on a hot plate (~40 °C) for 10 min. Then, the CNC and Alg solutions were mixed, followed by heating (~40 °C) and stirring on a hot plate for 10 min. The mixture solution was poured onto a Petri dish and air-dried at RT for 12 h to remove excess dH_2_O. This solution was immersed in 2 wt % CaCl_2_ solution for about 5 min. The CNC/Alg hydrogel film that formed was washed and rinsed with dH_2_O to remove excess CaCl_2_. For the formation of the bioAgNP/CNC/Alg hydrogel film, about 20 mg of bioAgNPs was added to the CNC solution and homogenized for 5 min before being mixed with the pre-heated Alg solution. The subsequent steps were the same as in the previously mentioned technique. A schematic diagram for the hydrogel film’s formation is presented in Figure 12.

#### 3.2.11. Fourier Transform Infrared Spectroscopy (FTIR) Analysis

A sample for FTIR analysis was sent to the Division of Instruments and MUPA, School of Chemical Sciences, Universiti Sains Malaysia. The analysis of the bioAgNPs, the CNC/Alg hydrogel film, and the bioAgNP/CNC/Alg hydrogel film was performed using a PerkinElmer Frontier™ FTIR spectroscope in the range of 600 to 4000 cm^−1^ with resolutions of 1 cm^−1^ and 4 cm^−1^. Before being subjected to analysis, the tested hydrogels were thawed briefly and then cut into small and thin films.

#### 3.2.12. Disc Diffusion Test of the bioAgNP/CNC/Alg Hydrogel Film

Initially, an overnight culture of *P. aeruginosa* USM-AR2 and MRSA was transferred to 5 mL of 0.85% sterile NaCl solution. The turbidity was adjusted using 0.5 McFarland solution (~10^8^ CFU/mL) before being diluted to ~10^5^ CFU/mL. An MH agar plate was evenly divided into 3 quadrants, namely a test sample (R), a positive control (+), and a negative control (−). About 100 µL of bacterial suspension was spread evenly on an MH agar plate using a sterile cotton swab. To obtain a sterile hydrogel film, a cork borer with a 10 mm diameter was flame-sterilized before being applied to bioAgNP/CNC/Alg. Subsequently, the obtained hydrogel film disc was further sterilized using UV irradiation for 10 min. Then, a sterile CNC/Alg or bioAgNP-CNC/Alg hydrogel disc (R), a vancomycin or streptomycin susceptibility disc (+), and dH_2_O (−) were placed on their respective quadrants on the MH agar plate. This test was done in triplicate. Next, the agar plates were incubated at 37 °C for 24 h. The average diameter of the inhibition zone was measured for each sample.

#### 3.2.13. Statistical Analysis

The results represent the mean ± S.E. from three independent experiments. To compare treated and untreated cells, Student’s *t*-test was performed using GraphPad Prism 8.0.1 (Graphpad software, Inc., San Diego, CA, USA). One-way ANOVA was performed to determine whether differences in the inhibition of the tested compounds were statistically significant. Student’s *t*-test was used to compare the inhibition efficiency between pathogenic bacteria.

## 4. Conclusions

We have successfully synthesized bioAgNPs using *Streptomyces* sp. PBD-311B supernatant as shown by color changes and UV-Vis spectra in the range of ~440 nm. Based on the TEM images, the average size of the bioAgNPs is 69 ± 2 nm and the nanoparticles are spherical in shape. The XRD analysis confirmed the formation of crystalline silver. The FTIR results show the presence of protein capping at the bioAgNP surface, which could be attributed to the extracellular protein binding to the bioAgNPs. The MIC value of bioAgNPs against *P. aeruginosa* USM-AR2 and MRSA is 6.25 mg/mL and 3.13 mg/mL, respectively. In addition, the bioAgNPs displayed cytotoxic effects against cancer cells (DBTRG-0.5MG and MCF-7) and showed minimal effects against normal cells (SVG-p12 and MCF-10A), conferring selective toxicity. Interestingly, the bioAgNPs still exhibited inhibition activity when incorporated in a CNC/Alg hydrogel film. It was also found that the bioAgNP-CNC/Alg hydrogel film displayed a minimal or slow release of bioAgNPs owing to the intermolecular interaction and the hydrogel film’s properties. Overall, bioAgNP-CNC/Alg is a promising antibacterial hydrogel film that inhibits the pathogenic bacteria *P. aeruginosa* and MRSA. Its application should be further evaluated for the inhibition of cancer cells. It shows benefits for the surgical resection of a tumor to avoid postoperative wound infections and tumor recurrence at the surgical site.

## Figures and Tables

**Table 1:** MIC values for the bioAgNPs and antibiotics against MRSA and *P. aeruginosa* USM-AR2.

Tested Bacteria	MIC Value (mg/mL)		
	bioAgNPs	Ampicillin	Streptomycin
MRSA	3.13 ± 0	0.016	-
*P. aeruginosa* USM-AR2	6.25 ± 0	-	0.016

**Table 2:** The half-maximal lethal concentration (LC_50_) values for DBTRG-05MG, SVGp12, MCF7, and MCF10A after treatment with various concentrations of bioAgNPs within 24–72 h. LC_50_ values were estimated using non-linear regression.

Treatment (h)	Cell Line/LC_50_ Value (µg/mL)			
	DBTRG-05MG	SVGp12	MCF7	MCF10A
24	194.65	>200 (n.d)	194.53	>200 (n.d)
48	172.66	>200 (n.d)	172.66	>200 (n.d)
72	103.27	>200 (n.d)	103.27	>200 (n.d)

n.d., not determined within the concentration range used.

**Table 3:** Comparison of the diameter of the inhibition zone between the bioAgNP-CNC/Alg hydrogel film and the CNC/Alg hydrogel film against *P. aeruginosa* USM-AR2 and MRSA. The values presented are the means ±S.E. The difference in inhibition between the tested compounds was considered statistically significant according to one-way ANOVA at *p* < 0.05.

Tested Compound	Diameter of the Inhibition Zone (mm)	
	*P. aeruginosa* USM-AR2	MRSA
CNC/Alg	0	0
bioAgNP-CNC/Alg	13 ± 0.7	11± 0
streptomycin (+)	10 ± 0.9	-
vancomycin (+)	-	17 ± 0
dH_2_O (−)	0	0

## Data Availability

The data presented in this study are available on request from the corresponding author.

## Sample Availability

Samples of the bioAgNP and bioAgNP/CNC/Alg are available from the authors.
